# Schisandrin B regulates the SIRT1/PI3K/Akt signaling pathway to ameliorate Ang II-infused cardiac fibrosis

**DOI:** 10.22038/ijbms.2025.83918.18160

**Published:** 2025

**Authors:** Xiaogang Zhang, Mengqing Shi, Zhongying Xing, Jie Su, Yuying Gu, Zhongping Ning

**Affiliations:** 1 Department of Cardiology, Shanghai Pudong New Area Zhoupu Hospital (Shanghai Health Medical College Affiliated Zhoupu Hospital) Shanghai; 2 201318, China; 3 Department of General Medicine, Shanghai University of Traditional Chinese Medicine (SHUTCM), Shanghai 201203, China; 4 Department of Cardiology, People’s Hospital of Shache County, Xinjiang, 844700, China

**Keywords:** α-SMA, Angiotensin II, Collagen I, Collagen III, PI3K/Akt, Sirtuin 1

## Abstract

**Objective(s):**

Schisandrin B (SchB), extracted from *Schisandra chinensis*, has antimicrobial and anti-inflammatory effects. The study aimed to investigate SchB’s possible defense against angiotensin II (Ang II)-infused cardiac fibrosis and its molecular processes.

**Materials and Methods:**

An equivalent volume of saline or Ang II (2.0 mg/kg/day, HY-13948, MedChemExpress) was administered subcutaneously to male C57BL/6 mice aged between 8 and 10 weeks. SchB (30 mg/kg/day, HY-N0089, MedChemExpress) was given via intraperitoneal injection two hours before Ang II infusion for 28 days. Comprehensive morphological, histological, and biochemical analyses were conducted. We evaluated the mRNA and protein expression levels using western blot and RT-qPCR techniques.

**Results:**

SchB treatment improves heart disease in Ang II-induced mice. SchB markedly lowered serum levels of cardiac fibrosis-related markers, including cTnI, cTnT, ANP, and BNP. In addition, SchB elevated sirtuin 1 (SIRT1) expression while reducing α-SMA, TGF-β1, collagen I, collagen III, and CTGF *in vivo*. Furthermore, SchB inhibited the migration of Ang II-infused rat cardiac fibroblasts. SchB increased SIRT1 expression while decreasing TGF-β1, α-SMA, collagen I, and collagen III, whereas EX-527, an inhibitor of SIRT1, recovered their activities *in vitro*. Furthermore, SchB elevated SIRT1 expression while lowering the expressions of p-PI3K (p85, Tyr458) and p-Akt (Ser473) proteins.

**Conclusion:**

Our results suggest that SchB regulates the SIRT1/PI3K/Akt pathway to prevent Ang II-infused cardiac fibrosis.

## Introduction

Myocardial fibrosis is caused by a rise in the production of extracellular matrix elements due to prolonged myocardial stress. It may alter blood flow pathways, stiffen the left ventricle, and deteriorate both systolic and diastolic functions (1-3). The onset of fibrosis has been associated with heightened risks of cardiovascular and overall mortality (4). Presently, no evidence-based pharmacological treatments exhibit significant effectiveness against fibrotic conditions, mainly due to the unidentified etiology of cardiac fibrosis. Furthermore, our comprehension of cardiac fibrosis mechanisms is predominantly derived from *in vitro* cell culture models or genetically modified murine models exhibiting heart cell-specific alterations (5).

The development of cardiovascular disorders is significantly influenced by the abnormal initiation of the renin-angiotensin system (RAS). Heart tissue and systemic circulation generate the primary RAS effector peptide, angiotensin II (Ang II). Ang II is related to the onset and progression of various cardiac dysfunctions, including diabetic cardiomyopathy, myocardial infarction, and alcoholic cardiomyopathy (6-8). Reactive oxygen species (ROS) are produced when Ang II attaches to its primary receptor, the Ang II receptor type 1 (AT1), initiating nicotinamide adenine dinucleotide phosphate (NADPH) oxidase (9). Oxidative stress arises when the levels of ROS surpass the body’s natural anti-oxidant defenses. Cardiomyocyte apoptosis or necrosis may result from the rapid activation of apoptosis-signaling pathways caused by the oxidative stress infused by Ang II. Heart failure and ventricular remodeling are ultimately caused by this mechanism (10). The mitogen-activated protein kinase pathway, nuclear factor-kappa B (NF-kB), and the epidermal growth factor receptor are among the routes via which elevated ROS levels can stimulate apoptosis, cardiac inflammation, ventricular remodeling, and hypertrophy (11, 12).

A highly abundant nuclear protein, sirtuin 1 (SIRT1), is present in many body tissues. SIRT1 regulates the activity of nuclear transcription factors by migrating from the cytoplasm to the nucleus upon activation (13). SIRT1 deacetylates histones to alter gene transcription. Additionally, deacetylating several non-histone proteins helps to prevent oxidative stress, aging, apoptosis, cellular proliferation, energy metabolism, differentiation, and other physiological and pathological processes (14, 15). An earlier study suggested that activating SIRT1 might prevent immediate kidney damage and lessen the inflammatory reaction stimulated by sepsis (16). According to early studies, system Xc**¯** expression and activity are regulated by the Nrf2 signal channel (17). Furthermore, a critical mechanism that significantly influences cellular functions is the phosphoinositide 3-kinase/AKT (PI3K/AKT) signaling pathway (18). This pathway, which is called after two crucial genes, PI3K and AKT, functions by phosphorylating downstream substrates (19). This pathway’s primary roles include boosting angiogenesis in response to external cues, enabling metabolism, encouraging cellular survival, and stimulating growth (20, 21). Nevertheless, how the SIRT1/PI3K/Akt pathway affects Ang II-infused cardiac fibrosis is unclear.

Schisandrin B (SchB), the principal active ingredient of *Schisandra chinensis*, is a time-honored remedy in traditional Chinese herbal medicine. This unique compound is a natural therapeutic agent with numerous beneficial effects. These effects include its anti-inflammatory properties, resistance to oxidative stress, and defense against microbial pathogens (22, 23). SchB, a natural, non-enzymatic anti-oxidant, has also been reported to be safe, cost-effective, and suitable for managing various health disorders. More recently, studies have demonstrated that SchB can reduce inflammation by preventing the NF-**κ**B signaling pathway and attenuating the pro-inflammatory cytokine levels, such as TNF-α, IL-1β, and IL-6.

Moreover, early research stated that SchB offers protective effects against inflammatory injuries in conditions such as inflammatory bowel disease (IBD) and acute lung injury (24, 25). In addition, SchB has been documented to mitigate the occurrence of nephrolithiasis by inhibiting inflammation and the mechanisms of ferroptosis (26). Furthermore, SchB has been noted to alleviate arthritis induced by adjuvants by attenuating inflammatory processes and oxidative stress (27). Additionally, previous investigations showed that SchB can inhibit the onset of diabetes by enhancing insulin secretion (28). However, It is yet unknown how the SchB could affect Ang II-induced myocardial fibrosis by regulating the SIRT1/PI3K/Akt pathway. Therefore, we conducted the current investigation to understand further the molecular process behind SchB’s defensive role against Ang II-infused cardiac fibrosis.

## Materials and Methods

### Animals and treatment

We considered male C57BL/6 mice aged 8 to 10 weeks and placed them in a pathogen-free (SPF) environment. They were then given either saline or a dosage of 2.0 mg/kg/day of Ang II (HY-13948, MedChemExpress) subcutaneously in mice using Model 2004, Alzet osmotic mini-pumps from the USA. The same volume of saline or a dosage of 30 mg/kg/day of SchB (HY-N0089, MedChemExpress) was given intraperitoneally. Three groups of mice, each consisting of eight mice, were randomly assigned: (1) Control group: mice had a subcutaneous saline infusion and injected intraperitoneally with saline. (2) Ang II group: mice had an intraperitoneal saline injection and were infused with Ang II. (3) SchB group: mice had an intraperitoneal SchB injection and subcutaneous induction of Ang II. On days 0, 7, 14, 21, and 28, a Softron BP98A tail-cuff device from Tokyo, Japan, was carried out to assess the mouse’s systolic blood pressure (SBP).

### Echocardiography

The heart function of mice was evaluated using two-dimensional echocardiography with the Vevo3100 model, a Small Animal Ultrasound Imaging System from Canada. Using M-mode imaging, we measured parameters including heart rate (HR), ejection fraction (EF), fractional shortening (FS), left ventricular end-systolic diameter (LVESd), left ventricular posterior wall thickness (LVPWth), and left ventricular end-diastolic diameter (LVEDd) (29).

### Sample collection

Following a 28-day Ang II infusion, each mouse was weighed, anesthetized, and had serum collected. Measurements of body weight (BW), tibia length (TL), and heart weight (HW) were taken to determine the HW/BW and HW/TL ratios. Histological analysis samples were treated with 4% paraformaldehyde, while RT-qPCR and western blot samples were snap-frozen in liquid nitrogen.

### Biochemical analysis

On day 28 following the initial Ang II infusion, the mice’s serum was taken to measure biochemical markers, such as the activity of mouse brain natriuretic peptide (BNP; ELISA kit: E-EL-M0204c, Elabscience, China), mouse atrial natriuretic peptide (ANP; ELISA kit: E-EL-M0166c, Elabscience, China), mouse cardiac troponin I (cTnI) (ELISA kit: SEKM-0153, Solarbio, China), and mouse cardiac troponin T (cTnT) (ELISA kit: SEKM-0150, Solarbio, China).

### Histology

To evaluate cardiac fibrosis, ventricular muscle tissue obtained from mice was labeled with Masson’s trichrome (D026-1-1; Nanjing Jiancheng Bioengineering Institute). The degree of fibrosis was assessed by calculating the blue-stained area ratio to the total myocardium area. Immunohistochemistry was performed on ventricular muscle tissue using the SIRT1 antibody (sc-74465, mouse monoclonal, Santa Cruz) (30).

### Isolation of rat cardiac fibroblasts

Pentobarbital sodium (60 mg/kg) was injected intraperitoneally to anesthetize the newborn SD rats. Once the heart had been separated, the ventricles were sliced and placed in a sterile culture plate. The cardiac tissues were divided into 1 mm³ pieces and passed through a 200-mesh filter. At 37 °C and 5% CO₂, the cardiac fibroblasts were cultivated in DMEM containing 10% FBS. The cardiac fibroblasts were photographed under an inverted microscope and recognized using Vimentin antibody and DAPI staining under a fluorescence microscope (31).

### MTT assay

Seeding in 96-well plates was done at a density of 2 × 10³ cells per well, and the cells were either treated with or without an inhibitor. After 48 hr, 20 µl of MTT (Solarbio, Beijing, China) was applied, and the cells were grown for four hours at 37 °C. After adding 200 µl of DMSO to each well, the absorbance at 570 nm was measured to determine the number of viable cells.

### Cell migration assay

The Transwell assay was carried out to assess the migratory ability of ventricular tissues. Cells were cultured in the higher chamber of Transwell plates (8 µm, 1 × 10⁵ cells/ml) in 100 µl of DMEM without FBS. The lower compartment had 600 μl of DMEM containing 10% FBS. A cotton swab was used to remove the cells from the upper part following a 72-hour incubation period. The migrating cells were then labeled with 0.1% crystal violet. Under a light microscope with a magnification of ×200, the average number of migrated cells was determined by counting the number of labeled moving cells from five fields (32).

### Cellular immunofluorescence

As reported previously, the immunofluorescence technique was used (33). Following three PBS washes, α-SMA (ab7817, Abcam, UK) and vimentin (ab92547, Abcam, UK) antibodies were applied to frozen slices of mouse ventricular tissue. After counterstaining the cells with DAPI, we observed them using model IX51, an inverted Olympus microscope from Japan. The procedures for vimentin and α-SMA were completed based on the developer’s references.

### RT-qPCR

We isolated total RNA from ventricular tissues using commercially available kits (TRIzol, Invitrogen, USA). Subsequently, we conducted a real-time quantitative PCR (RT-qPCR) reaction to amplify the mRNA using the TaKaRa SYBR Green reagent from Japan and an ABI Prism 7700 model real-time PCR machine from the USA. The 2^-^^ΔΔ^^Ct^ formula was employed to assess the expression of relative genes, with GAPDH serving as an internal control (34). We designed primer sequences using the NCBI Primer-BLAST tool, as shown in Table 1.

### Western blotting

Following cell lysis, we isolated the protein samples using the commercially available RIPA lysis buffer (Beyotime Biotechnology) obtained from Shanghai, China. We then used a Beyotime BCA kit to ascertain the protein concentrations. After that, 40 μg of protein was mixed with the Beyotime loading buffer and denatured in a hot water bath for three minutes. After bromophenol blue was prolonged to the separating gel, electrophoresis began at 80 V for 30 min. A second phase was conducted at 120 V for one to two hours.

After 60 mi in the ice bath at 300 mA, the proteins were transferred to membranes. Following a one- to two-minute rinse with a washing solution, the membranes were either sealed overnight at 4 °C or inactivated for an hour at 20 °C. For one hour at 20 °C, primary antibodies against SIRT1 (1:400, sc-74465, mouse monoclonal, Santa Cruz), α-SMA (1:500, ab7817, mouse monoclonal, Abcam), p-PI3K (p85, Tyr458) (1:400, ab278545, rabbit monoclonal, Abcam), t-PI3K (1:500, ab302958, rabbit monoclonal, Abcam), p-Akt (Ser473) (1:400, ab81283, rabbit monoclonal, Abcam), t-Akt (1:400, ab8805, rabbit polyclonal, Abcam), and GAPDH (1:1000, ab8245, mouse monoclonal, Abcam) were applied to the membranes on a shaking table. The membranes were exposed to the secondary antibody at 20 °C for one hour and then again for ten minutes, each time using a washing solution. Finally, the membranes were immersed in the developing solution and examined using a Gel Doc XR model Bio-Rad chemiluminescence imaging analysis equipment (31).

### Statistical analysis

The findings of at least three tests were represented using the average divided by the standard deviation (SD). GraphPad Prism 9.0 was used to conduct the statistical comparison. Multiple group differences were assessed using the *post hoc* Tukey test and one-way ANOVA. A *P*-value<0.05 was deemed to indicate statistical significance.

## Results

### SchB improves cardiac dysfunction of Ang II-infused mice

**Figure 1:**
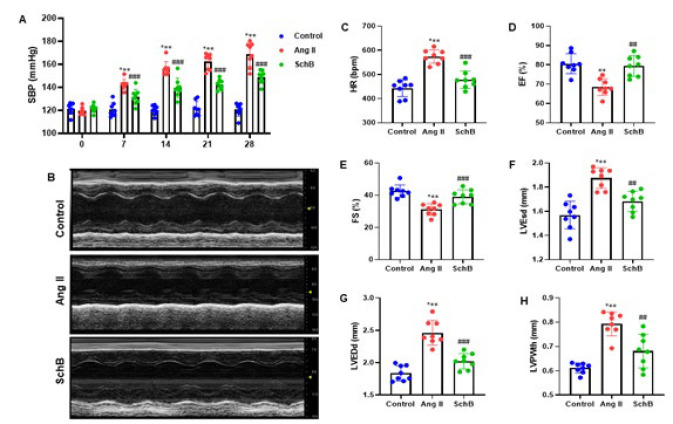
SchB improves cardiac dysfunction of Ang II-infused mice

In this experiment, we examined SchB’s beneficial effect on heart disease of Ang II-induced mice. For 28 days, mice received subcutaneously intraperitoneal injections of SchB (30 mg/kg/day) and an infusion of Ang II (2.0 mg/kg/day). Mice were given Ang II, and their systolic blood pressure (SBP) was examined at 0, 7, 14, 21, and 28 days. The results showed that Ang II induction elevated SBP, which was reduced by SchB (Figure 1A). Figure 1B represents images of M-mode echocardiography in mice of each group. SchB showed markedly improved echocardiographic parameters, including HR, LVESd, LVEDd, and LVPWth decreased and increased EF and FS rates (Figure 1C-D). These results suggest that SchB treatment improves cardiac disorder in Ang II-induced mice.

### SchB attenuated Ang II-infused cardiac fibrosis

**Figure 2:**
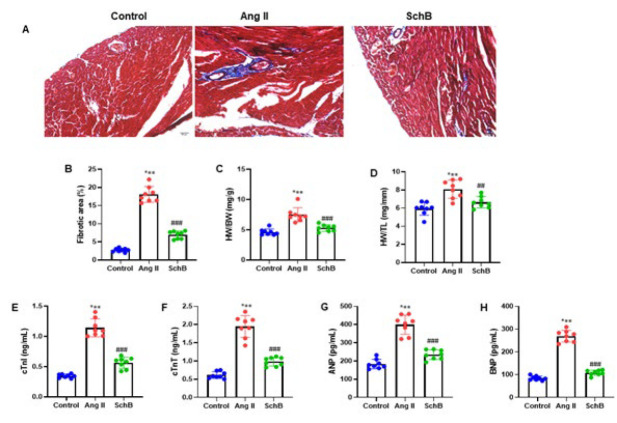
SchB suppressed cardiac fibrosis in Ang II-induced mice

Two hours before the Ang II infusion began, a 2.0 mg/kg/day dose of Ang II was subcutaneously infused, and either a 30 mg/kg/day dose of SchB or an equivalent amount of saline was administered intraperitoneally to examine the effect of SchB on Ang II-induced cardiac fibrosis and molecular expression. SchB dramatically lowered the impact of Ang II, which greatly enhanced the renal fibrotic region (Figure 2A and B). The HW/BW and HW/TL ratios in mice were calculated to assess cardiac hypertrophy. The results showed that Ang II induction markedly elevated the HW/BW and HW/TL ratios in mice, while SchB significantly reduced these ratios (Figure 2C and D). ANP, BNP, cTnI, cTnT, and other indicators linked to cardiac fibrosis were evaluated in serum using ELISA. The findings showed that whereas SchB may have decreased serum levels of cTnI, cTnT, ANP, and BNP, Ang II considerably raised them (Figure 2E-H). The aforementioned outcomes indicated that in mice given Ang II, SchB prevented myocardial fibrosis.

### SchB elevated SIRT1 expression in heart tissue of Ang II-induced mice

**Figure 3:**
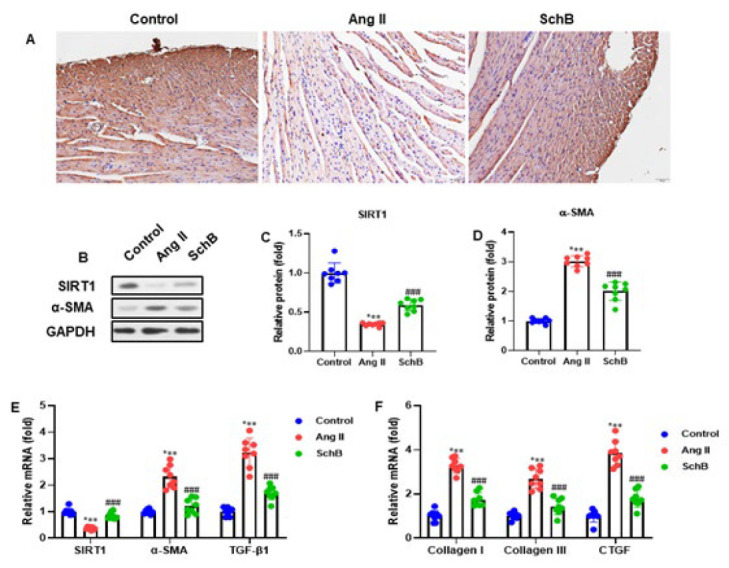
SchB enhanced SIRT1 expression in cardiac tissue of Ang II-infused mice

For 28 days, mice were given Ang II with or without SchB (30 mg/kg/day) to inspect any possible effects of SchB on SIRT1 expression in the heart tissue of Ang II-induced mice. Ang II markedly decreased the SIRT1 expression. Meanwhile, SIRT1 expression was markedly elevated by SchB (Figure 3A). Additionally, we performed a western blot analysis to estimate the SIRT1 and α-SMA protein expression. While Ang II induction raised the amount of α-SMA, we observed that it decreased the expression of SIRT1. SIRT1 and α-SMA expression patterns in Ang II-infused mice were reversed by SchB therapy (Figure 3B). Following the measurement of the band intensity, we noticed that SIRT1 and α-SMA exhibited identical expression trajectories (Figure 3C, D). Protein expression was normalized to GADPH. Moreover, collagen I, collagen III, TGF-β1, SIRT1, α-SMA, and CTGF mRNA expressions were also assessed using RT-qPCR analysis. Ang II infusion was shown to increase α-SMA, TGF-β1, collagen I, collagen III, and CTGF while decreasing SIRT1 mRNA levels. SchB treatment elevated the SIRT1 and reduced the α-SMA, TGF-β1, collagen I, collagen III, and CTGF in Ang II-induced mice (Figure 3E, F). These findings showed that, in the heart tissue of mice infused with Ang II, SchB therapy increased SIRT1 expression.

### SchB inhibits Ang II-infused migration of rat cardiac fibroblasts

**Figure 4:**
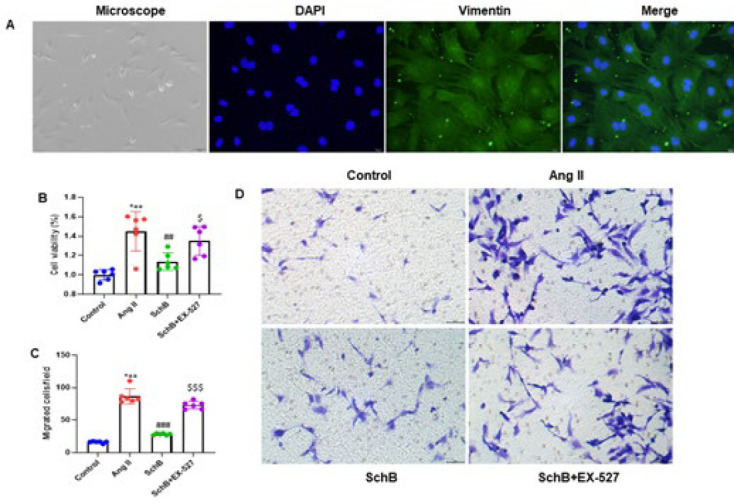
SchB inhibits Ang II-induced migration of rat cardiac fibroblasts

Primary cardiac fibroblasts were obtained from the ventricular myocytes of neonatal SD rats to assess the possible effects of SchB on Ang II-infused migration of rat cardiac fibroblasts. Vimentin and DAPI staining verified their identification. Cardiac fibroblasts were identified by immunofluorescence analysis (Figure 4A). A SIRT1 inhibitor, EX-527 (10 μM), and SchB (20 μM) were administered to cardiac fibroblasts as pretreatment for two hours. The cells were treated for 48 hr with Ang II (1 μM). The MTT procedure was used to evaluate cell viability. We found that Ang II induction increased the rate of cell proliferation, which was reduced by SchB treatment, and a SIRT1 inhibitor, EX-527, restored the rate of cell proliferation (Figure 4B). We used the Transwell test to measure cell migration. Six randomly selected fields were used to determine the average quantity of migrated cells per field. The results indicate that the migration ability was stimulated by Ang II infusion, while the SchB treatment lowered the migration capacity. On the other hand, the EX-527 treatment recovered the migration ability (Figure 4C). Furthermore, we investigated the migration ability of the cells under the inverted microscope. We observed the same trends in the migration ability of the Ang II-infused cardiac fibroblasts in rats (Figure 4D). The findings imply that SchB inhibited the migration of rat fibroblasts from the heart-infused by Ang II. 

### SchB inhibited the differentiation of cardiac fibroblasts stimulated by Ang II

**Figure 5:**
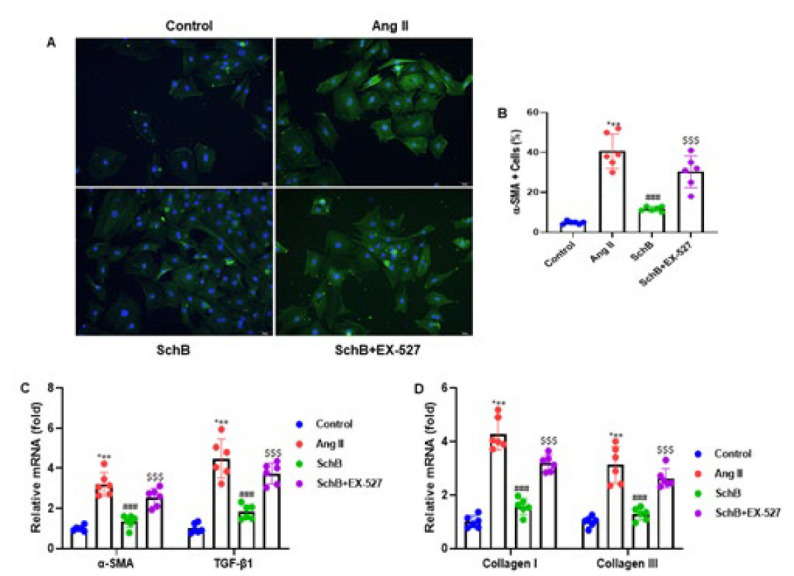
SchB suppresses the Ang II-induced diﬀerentiation of rat cardiac fibroblasts

In order to assess SchB’s possible role in the differentiation of cardiac fibroblasts triggered by Ang II, we carried out the cellular immunofluorescence in cardiac fibroblasts using α-SMA antibody. The experimental outcomes indicated that Ang II infusion raised the differentiation of cardiac fibroblasts, while SchB treatment reduced cellular differentiation. On the other hand, the Ex-527 treatment restored the increasing differentiation of the cardiac fibroblasts (Figure 5A). Further, we quantified the α-SMA+ cells to the DAPI+ cells. We found the same trends of cellular differentiation pattern (Figure 5B). mRNA α-SMA, TGF-β1, collagen I, and collagen III expression were assessed using RT-qPCR. Ang II infusion elevated the α-SMA, TGF-β1, collagen I, and collagen III mRNA expressions, while SchB treatment lowered the expression levels. On the other hand, the Ex-527 treatment restored the increasing α-SMA, TGF-β1, collagen I, and collagen III mRNA expression (Figure 5C, D). The experimental data demonstrated that SchB suppressed the Ang II-induced differentiation of cardiac fibroblasts.

### SchB modulated the SIRT1/PI3K/Akt pathway in Ang II-infused differentiation of cardiac fibroblasts

**Figure 6:**
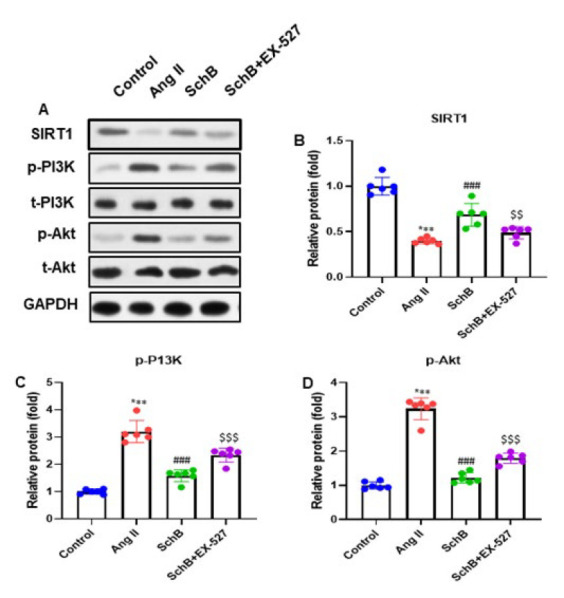
SchB modulates the SIRT1/PI3K/Akt pathway in Ang II-induced diﬀerentiation of rat cardiac fibroblasts

The protein expression of the SIRT1/PI3K/Akt pathway was assessed by western blotting to evaluate the possible impact of SchB on this pathway in the Ang II-infused differentiation of cardiac fibroblasts. The study outcomes indicated that Ang II induction reduced the SIRT1 protein level and increased p-PI3K (p85, Tyr458) and p-Akt (Ser473) protein expressions, while SchB treatment reversed the protein expression levels. On the other hand, the EX-527 treatment restored the protein expression pattern (Figure 6A). After quantifying the proteins of SIRT1, p-PI3K (p85, Tyr458), and p-Akt (Ser473) (normalized to GADPH protein), we observed the consistent trends of SIRT1, p-PI3K (p85, Tyr458), and p-Akt (Ser473) protein expressions (Figure 6B-D). The results indicated that SchB regulated the SIRT1/PI3K/Akt pathway in Ang II-infused differentiation of cardiac fibroblasts.

## Discussion

The current investigation assessed the impact of SchB on heart fibrosis infused by Ang II and the underlying molecular mechanisms involved. The investigation outcomes indicated that SchB therapy improved heart function and increased SIRT1 expression in the cardiac tissue of mice induced with Ang II while also reducing cardiac fibrosis. Additionally, SchB inhibited the migration and differentiation of rat cardiac fibroblasts infused by Ang II. Furthermore, SchB was found to regulate the SIRT1/PI3K/Akt pathway during the differentiation of cardiac fibroblasts triggered by Ang II. These results imply that SchB may modulate the SIRT1/PI3K/Akt pathway to prevent Ang II-induced cardiac fibrosis.

One dangerous arrhythmia that can result in death is atrial fibrillation (AF). The improvement and progression of AF-related diseases are influenced by various factors (30). The most frequent cause of structural remodeling and the duration of AF in patients is atrial fibrosis. Structural, electrical, and autonomic changes in the left atrium (LA) are related to AF (35, 36). Atrial fibrosis in the LA facilitates AF’s development, progression, and maintenance (37). Cardiac fibrosis is a collective characteristic in human AF patients and experimental models (38). In this investigation, we used a mouse model to inspect the effect of SchB on Ang II-induced cardiac fibrosis and the related molecular entities. Consistent with earlier studies, the outcomes indicated that SchB therapy lowered Ang II-infused cardiac fibrosis and altered its associated molecular markers, such as α-SMA, collagen I, and collagen III (39).

In cardiac tissues, angiotensin II (Ang II) is the primary modulator of oxidative stress, increasing the generation of ROS in the vascular system by initiating membrane-associated NADPH oxidase (NOX), which leads to endoplasmic reticulum stress and mitochondrial oxidative stress (40). The presence of ROS has been implicated in the pathogenesis of cardiac fibrosis, which facilitates the onset and advancement of AF. This phenomenon initiates a significant positive feedback mechanism, culminating in the manifestation of AF and subsequently exacerbated fibrosis (41). Previous investigations have demonstrated that the attenuation of Ang II-infused proarrhythmic responses in AF can be achieved by targeting the oxidative loss of CaMKII in oxidation-resistant CaMKII MMVV murine models (42). The study’s authors reported that CaMKII operates as an initial signaling entity for ROS, a process that NOX can activate (43). However, the present investigation showed that Ang II raised the rate of fibrotic area, CTnl, CTnT, ANP, BNP, α-SMA, collagen I, collagen III, CTGF, and TGF-β1 while reducing SIRT1, which was reversed by SchB therapy in the Ang II-induced mice. In addition, cell proliferation, migration, α-SMA, TGF-β1, collagen I, collagen III, p-PI3K (p85, Tyr458), and p-Akt (Ser473) were increased, and SIRT1 was decreased by Ang II infusion in the rat cardiac fibroblasts where reversed by SchB treatment. On the other hand, EX-527, an inhibitor of SIRT1, recovered their activities.

In traditional Chinese herbal medicine, SchB, an active phytochemical derived from *Schisandra chinensis*, has long been used as a medicinal agent. This bioactive substance has several beneficial characteristics and is a natural medicinal agent. Its anti-inflammatory effectiveness, ability to combat oxidative stress, and protection against microbial infections are among these qualities (22, 23). As a natural, non-enzymatic anti-oxidant, SchB is regarded as safe and affordable, making it a viable option for treating a range of illnesses. According to recent empirical research, SchB can lessen inflammatory reactions by lowering pro-inflammatory cytokine levels and blocking the NF-κB signaling pathway. It has been demonstrated that SchB offers protective advantages against inflammatory injuries in diseases such as acute lung damage and inflammatory bowel disease (IBD) (24, 25). By suppressing inflammatory reactions and ferroptosis-related processes, SchB has also been shown to reduce the incidence of nephrolithiasis (26). Moreover, SchB has been shown to mitigate adjuvant-induced arthritis by lowering oxidative stress and inflammatory responses (27). Additionally, by increasing insulin production, SchB has been shown to delay the onset of diabetes (28) significantly. However, our study showed that SchB alleviated Ang II-infused cardiac fibrosis by modulating the SIRT1/PI3K/Akt pathway.

**Table 1:** List of oligonucleotide primer sequences for mouse and rat genes used in this research

Genes	Forward primer (5′-3′)	Reverse primer (5′-3′)
Mouse SIRT1	TCGGCTACCGAGGTCCATA	AACAATCTGCCACAGCGTCA
Mouse α-SMA	TCCTGACGCTGAAGTATCCGATA	GGCCACACGAAGCTCGTTAT
Mouse TGF-β1	AGCAACAATTCCTGGCGTTACCTT	CCTGTATTCCGTCTCCTTGGTTCAG
Mouse Collagen I	GCTCCTCTTAGGGGCCACT	CCACGTCTCACCATTGGGG
Mouse Collagen III	TCCCCTGGAATCTGTGAATC	TGAGTCGAATTGGGGAGAAT
Mouse CTGF	CCAGACCCAACTATGATGCG	GTGTCCGGATGCACTTTTTG
Rat α-SMA	CTATTCCTTCGTGACTACT	ATGCTGTTATAGGTGGTT
Rat TGF-β1	AACAATTCCTGGCGTTACCT	GCCCTGTATTCCGTCTCCTT
Rat Collagen I	GCCTCAGCCACCTCAAGAGA	GGCTGCGGATGTTCTCAATC
Rat Collagen III	CCAGGACAAAGAGGGGAACC	CCATTTCACCTTTCCCACCA
Mouse GAPDH	ACTCCACTCACGGCAAATTC	TCTCCATGGTGGTGAAGACA
Rat GAPDH	CCCCCAATGTATCCGTTGTG	TAGCCCAGGATGCCCTTTAGT

α-SMA: α-smooth muscle actin; TGF-β1: transforming growth factor-β1; CTGF: connective tissue growth factor; GAPDH: Glyceraldehyde-3-phosphate dehydrogenase.

(A) The mice's blood pressure (SBP) was measured at 0, 7, 14, 21, and 28 days after Ang II infusion. (B) Representative images of M-mode echocardiography in mice of each group. SchB markedly improved the echocardiographic parameters and decreased (C) HR, increased (D) EF and (E) FS, decreased (F) LVESd, (G) LVEDd, and (H) LVPWth. Data are presented as mean ± SD (n=8 in each group). ***P<0.001 vs Control group; ##*P*<0.01, ###*P*<0.001 vs Ang II group. Ang II, angiotensin II; SchB, Schisandrin B; SBP, systolic blood pressure; HR: heart rate; EF: ejection fraction; FS: fractional shortening; LVESd: left ventricular end-systolic diameter; LVEDd: left ventricular end-diastolic diameter; LVPWth: left ventricular posterior wall thickness.

(A) Ventricular muscle tissue was stained with Masson trichrome (Magnification 200×). (B) Cardiac fibrosis was quantified by calculating the percentage of the fibrotic area. (C, D) Cardiac hypertrophy was evaluated by calculating the HW/BW and HW/TL ratios in mice. The serum levels of cardiac fibrosis-related markers, including (E) cTnI (ng/ml), (F) cTnT (ng/ml), (G) ANP (pg/ml), and (H) BNP (pg/ml), were measured by ELISA. Data are presented as mean ± SD (n=8 in each group). ***P<0.001 vs Control group; ###*P*<0.001 vs Ang II group. HW: heart weight; BW: body weight; TL: tibia length; cTnI: cardiac troponin I; cTnT: cardiac troponin T; ANP: atrial natriuretic peptide; BNP: brain natriuretic peptide

(A) Representative images of immunohistochemistry in ventricular muscle tissue by SIRT1 (200×). (B) Western blot was applied to determine the protein expression of SIRT1 and α-SMA. (C, D) Quantification of protein bands of SIRT1 and α-SMA. RT-qPCR was applied to determine the mRNA expression of (E) SIRT1, α-SMA, TGF-β1, and (F) Collagen I, Collagen III, and CTGF. GADPH serves as an internal control. Data are presented as mean ± SD (n = 8 in each group). ***P<0.001 vs control group; ###*P*<0.001 vs Ang II group. α-SMA: α-smooth muscle actin; TGF-β1: transforming growth factor-β1; CTGF: connective tissue growth factor.

(A) Primary cardiac fibroblasts were stained by Vimentin and DAPI. (B) MTT was performed to evaluate cell viability. (C) Cell migration was assessed by Transwell assay. (D) Representative images of migrated cardiac fibroblasts under an inverted microscope. Data are shown as mean ± SD (n=6 per group) from three independent experiments. ***P<0.001 vs the control group; ##*P*<0.01, ###*P*<0.001 vs Ang II group; $*P*<0.05, $$$ *P*<0.001 vs SchB group. SchB: Schisandrin B

(A) Cellular immunofluorescence was applied to cardiac fibroblasts using α-SMA antibody (200×). (B) The α-SMA+ cells were quantified relative to DAPI+ cells. RT-qPCR was performed to determine the mRNA expression of (C) α-SMA and TGF-β1, and (D) Collagen I and Collagen III. GADPH served as an internal control. Data are shown as mean ± SD (n=6 per group). ***P<0.001 vs control group; ###*P*<0.001 vs Ang II group; $$$*P*<0.001 vs SchB group. α-SMA: α-smooth muscle actin; TGF-β1: transforming growth factor-β1; SchB: Schisandrin B

(A) Western blot was performed to determine the protein expression of SIRT1/PI3K/Akt pathway. Quantification of proteins (B) SIRT1, (C) p-PI3K (p85, Tyr458), and (D) p-Akt (Ser473) (normalized to GADPH protein). Data are presented as mean ± SD (n = 6 per group). ***P<0.001 vs control group; ###*P*<0.001 vs Ang II group; $$$*P*<0.001 vs SchB group. SchB: Schisandrin B; SIRT1: SchB elevated sirtuin 1; PI3K: phosphoinositide 3-kinase/AKT

## Conclusion

Our investigation examined the protective effects of SchB against cardiac fibrosis infused by Ang II using both *in vivo* and *in vitro* methods. The evidence from our studies shows that SchB treatment remarkably decreased Ang II-infused cardiac fibrosis by modulating the SIRT1/PI3K/Akt signaling pathway. In future research efforts, validating these findings in clinical and preclinical settings will be essential to support our conclusions further. 

## Data Availability

The datasets used and/or analyzed during the current study are available from the corresponding author upon reasonable request.

## Declaration

We have not used any AI tools or technologies to prepare this manuscript.

## Limitations

This investigation has some limitations. First, there was no dose-dependent evaluation of SchB’s efficacy. Second, the initial findings of this study have yet to be established in preclinical or clinical settings. Third, further research is needed to clarify the mechanisms contributing to SchB’s defensive impact against Ang II-infused cardiac fibrosis. Our study offers valuable insights into SchB’s protective effects against cardiac fibrosis despite its limitations.
